# Diseases of Women

**Published:** 1872-06

**Authors:** 


*Diseases of Women.* By T. GILLARD THOMAS, M. D. Third Edition. Enlarged and thoroughly revised. With two hundred and forty-six illustrations. Philadelphia: Henry C. Lea, 1872.

Thomas on diseases of women as offered to the profession in this, its third edition is the latest and best work in the English language, (or we believe any other language) upon this subject. We have taken great interest in carefully perusing its pages, and find that he has given his opinions without apparent bias, by what has been previously said either by himself or anybody else. If he wishes to condemn, he does so in a positive and manly way, and when he expresses opinions he carries conviction that he is correct.

The comprehensive character of work commends it to the profession, since nothing within its scope will be found wanting; it may truly be said to be complete in discussing every topic legitimately in this branch of practice. The older works upon diseases of women have proven almost obsolete, in the rapid process of this department of medicine. A few years makes it necessary to change much of the teaching or omit it altogether; revision of a work upon diseases of women has been a necessity. That it will be less necessary in the future, is quite improbable, since knowledge in this department is nearly all of it of comparatively recent date, and our present views and modes of proceedure will be modified by experience and by the correcting influences of time. Meanwhile the work of Prof. Thomas may be relied upon as containing what is known of the diseases of women.

